# Microscale Affinity Chromatography for Biointeraction Analysis: Strategies, Principles and Applications

**DOI:** 10.1002/jssc.70361

**Published:** 2026-02-08

**Authors:** David S. Hage, Nigar Sultana Pinky, B. K. Sajeeb, Md Masudur Rahman, Harshana Olupathage, Samiul Alim, Isaac Kyei, Zoe Zingler, Sanduni Heenkenda

**Affiliations:** ^1^ Department of Chemistry University of Nebraska‐Lincoln Lincoln Nebraska USA

**Keywords:** affinity microcolumn, binding studies, biointeraction analysis, kinetic studies, microscale affinity chromatography

## Abstract

The analysis of interactions between biological agents or with surrounding chemicals is important in many areas of modern biochemical, biomedical, and environmental research. Microscale platforms based on affinity chromatography have been shown to be a powerful set of tools for these studies. This approach makes use of an immobilized binding agent as a stationary phase in a microscale platform for either direct examination of the interactions of this agent with an applied target solute or as a secondary capture agent to probe a solution‐phase interaction. This review will examine the various platforms and strategies that have been used in microscale affinity chromatography, or µAC, to characterize and study biointeractions. The general principles of µAC and schemes based on this approach will be examined, along with applications of this technique. Examples of approaches that will be considered will include zonal and frontal analysis methods, as well as a variety of schemes by which µAC can be employed in kinetic studies. In each case, the theory and principles of these methods will be provided along with examples of their use in biointeraction studies.

## 1 Introduction

The analysis of biological and chemical interactions, or “biointeractions”, as part of molecular recognition is essential for understanding most processes in natural and living systems [1, 2, 3, 4, 5]. Examples include enzyme‐substrate binding, receptor–ligand signaling, antibody–antigen interactions, and gene regulation [2, 3, 4, 5, 6]. Information on these processes is important in studying disease mechanisms, developing new treatments for disease, and creating biosensors or analytical methods for applications such as disease detection and therapeutic monitoring [1, 2, 3, 4, 5, 6, 7, 8, 9].

Many techniques have been developed to study biointeractions [2, 3, 4, 5]. Traditional methods have included tools such as equilibrium dialysis and ultrafiltration, along with spectroscopic techniques based on absorbance, fluorescence, or surface plasmon resonance [2, 3, 4]. Other methods that have been employed for this purpose are calorimetry, biolayer interferometry, nuclear magnetic resonance spectroscopy, microscale thermophoresis, and liquid chromatography‐mass spectrometry [2, 9, 10, 11, 12, 13, 14, 15]. In addition, systems such as drug‐target binding have been examined by using computational methods (e.g., simulations of molecular docking and molecular dynamics) and proteomic techniques [2, 9, 12]. However, there is still a need for rapid, accurate, and precise approaches that can be used for biointeraction studies and that require only small amounts of samples and/or binding agents [2, 3, 4, 9].

**FIGURE 1:**
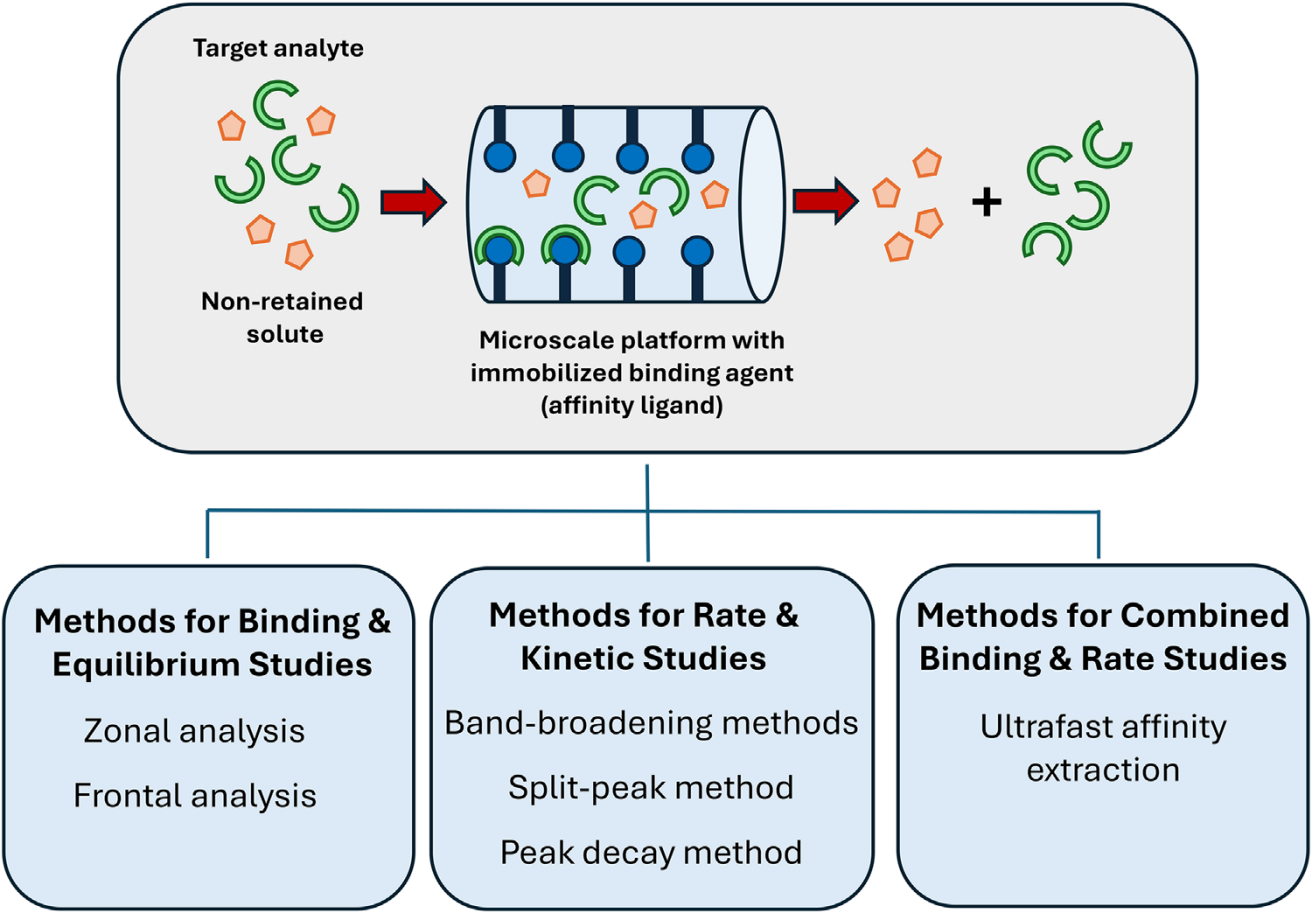
General scheme for microscale affinity chromatography (µAC) and examples of methods that can be used with this technique for the study of biointeractions.

There are various separation methods that may employ biological or natural binding agents and that can be used to investigate their interactions. For instance, affinity capillary electrophoresis (ACE) has been used for this purpose, as discussed in previous papers and reviews on this topic [4, 16, 17, 18, 19, 20, 21, 22, 23, 24, 25, 26, 27, 28, 29, 30]. Another example of such a method, and the focus of this review, is microscale affinity chromatography (µAC) [1, 2, 3, 4]. Affinity chromatography, as is illustrated in Figure 1, is a type of liquid chromatography in which a biologically‐related binding agent is immobilized onto a support and used as the stationary phase [1]. This immobilized agent, often referred to as the “affinity ligand,” can then be used either directly for biointeraction studies or as a secondary capture agent in such experiments [1, 2]. High‐performance affinity chromatography (HPAC) is a form of affinity chromatography in which the immobilized agent is used with an HPLC support and associated equipment [1, 2, 31, 32, 33, 34]. µAC, in turn, is a type of HPAC and affinity chromatography which employs columns with total volumes in the low‐to‐mid microliter range [9, 35, 36, 37, 38, 39, 40]. The use of microscale platforms in this latter method have been of great interest in recent years for the study of biointeractions due to their need for small amounts of binding agent and sample as well as their speed, ease of automation, and ability to be used with a wide range of techniques and detection modes [1, 4, 5].

This review will examine the formats and approaches that have been used for the study of biointeractions by µAC and associated platforms. Both zonal and frontal analysis methods in µAC will be discussed, as well as various approaches for combined binding and kinetic studies. Examples of applications will be provided from the pharmaceutical, biomedical, and environmental fields. The advantages and requirements of these technologies will also be examined.

## 2 General Principles of Microscale Affinity Chromatography

Both µAC and traditional affinity chromatography make use of the reversible interactions that can occur in biological systems [1, 41]. There are many binding agents that have been employed in these methods. Binding agents of biological origin have ranged from antibodies and antigens to enzymes, serum proteins, immunoglobulin binding‐proteins, nucleic acids, lipids, and carbohydrates [1, 2, 41]. It is also possible that the binding agent may be synthetic and/or a mimic of a biological ligand. Examples of this second group are aptamers, molecularly imprinted polymers, triazine dyes, metal ion chelates, and boronates [1, 41]. In principle, any of these agents can be used in µAC for either chemical analysis or biointeraction studies if they represent a system with moderate‐to‐strong binding and have reasonable selectivity for their binding partners [9, 35, 36, 37, 38, 39, 40].

The following model is often used in µAC and related methods (e.g., HPAC and traditional affinity chromatography) to describe the interaction of a given target analyte (A) with a binding agent or ligand for this target (L). If these two components have 1:1 binding, their interaction can be described by the following general reaction and set of equations [1, 2, 3, 4, 16].

(1) $$\text{A+L}\overset{k_{a}}{\underset{k_{d}}{⇌}}\text{A-L}$$

(2) $$\mathrm{where}\;\;\;K_{a}=\frac{\left[A-L\right]}{\left[A\right]\left[L\right]}\;\;\;\;\;\;\;\mathrm{or}\;\;\;\;\;K_{d}=\frac{\left[A\right]\left[L\right]}{\left[A-L\right]}$$

(3) $$K_{a}=\;\frac{k_{a}}{k_{d}}=\frac{1}{K_{d}}\;$$

In this scheme, A binds with L to create the reversible complex A‐L. The association equilibrium constant for this reaction is given by *K*_a_, and the reciprocal of this value (*K*_d_) is the corresponding dissociation equilibrium constant. Furthermore, the association and dissociation kinetics of this reaction are described by *k*_a_, the association rate constant, and *k*_d_, the dissociation rate constant. Related schemes and models can be developed to describe more complex interactions between A and L or other agents [16, 33].

There are several reasons why the types of binding agents and interactions that occur in affinity chromatography make this method especially appealing for the development of microscale separation and analysis platforms. First, there is the fact that most of the biological binding agents that are used in affinity chromatography are based on interactions that occur naturally in microscale systems, such as within or between cells, tissues, or organs in the body. Another key reason is the fact that the strong and selective binding for these interactions make it less essential in affinity chromatography to have long and efficient columns than in other types of chromatography. This can be illustrated by the general resolution equation of chromatography, as given below [42].

(4) $$\;\;\;\;R_{s}=\left[{\left(N\right)}^{1/2}/4\right]\left[\left(\alpha -1\right)/\alpha \right]\left[k_{2}/\left(1+k_{2}\right)\right]$$

In this equation, *R*_s_ is the resolution between two neighboring peaks, *N* is the number of theoretical plates measured for these peaks, α is the separation factor (i.e., ratio of the retention factors) for the two peaks, and *k*_2_ is the retention factor for the second of the two peaks. This relationship shows that the extent of resolution in chromatography is determined by the efficiency of the system and extent of peak‐broadening (*N*), the selectivity of the column in its retention of the compounds in these two peaks (α), and the overall degree of retention for these compounds (*k*_2_) [42]. In both affinity chromatography and µAC, the values of α and *k*_2_ are often quite large [41]. This means *N* can even have moderate or low values and still allow good resolution between peaks. As column length is one factor that determines *N*, this also means that much smaller columns can often be employed in µAC than in many other forms of liquid chromatography [9, 43, 44].

The effect of column size on efficiency and resolution in µAC can be illustrated as follows. First, suppose the same support and affinity ligand are packed within both a standard 25 cm × 4.6 mm inner diameter (I.D.) column for HPAC and a 5 mm × 4.6 mm I.D. column for µAC. In this situation, there is a 50‐fold difference in both the length and the total volume of these columns (i.e., 25 vs. 0.5 cm and 4.15 mL vs. 83 µL at an I.D. of 4.6 mm). The number of theoretical plates (*N*) in this case will also decrease by 50‐fold when going to the shorter column. However, the value of *R*_s_ in Equation (4), which is proportional to (*N*)^1/2^, will decrease by only about 7‐fold. The values of α and *k_2_* in Equation (4) will both be unaffected, as they are independent of column size [42]. Thus, if α and *k_2_* are large and provide a combined product on the right of Equation (4) that gives a value for *R*_s_ of ∼1.5 or greater, the overall resolution for the shorter µAC column should still be more than sufficient even as there is a reduction in the value of *N* [41, 42].

There are additional advantages for µAC that are related to column size. For instance, when moving from a packed 25 cm × 4.6 mm I.D. HPAC column to a packed 5 mm × 4.6 mm I.D. µAC column, the amount of support and affinity ligand that are required will decrease in proportion to the column volume (i.e., by 50‐fold in this case). As a specific example, a silica support that contains the immobilized protein bovine serum albumin (BSA; MW, 66 kDa) at a level of 40 mg protein/g support will have 75 mg of this protein (1.1 µmol) within a 25 cm × 4.6 mm I.D. column but only 1.5 mg (22 nmol) in a 5 mm × 4.6 mm I.D. column. If the inner diameter of the 5 mm column is reduced from 4.6 to 1.0 mm, the internal volume will now be only 4 µL and contain 70 µg (1.1 nmol) of the same protein. In addition, the column void time and backpressure will also decrease with the column length and volume, which can be useful when the goal is to create methods with small void times or low backpressures.

Various formats have been used to reduce the size of separation platforms that are used with immobilized binding agents in µAC [9]. As suggested in the previous paragraphs, initial work in this area began with a simple reduction in the dimensions used in a conventional HPLC column, such as a decrease in the column length, diameter, or a combination of these parameters [46, 47, 48, 49, 50, 51]. The result is often called an “affinity microcolumn” [9]. Some advantages of affinity microcolumns are their need for only small amounts of binding agent or sample, their good stability, and their compatibility with standard HPLC equipment and detection formats (e.g., absorbance, fluorescence, and mass spectrometry) [38, 47, 48, 49, 50, 51, 52, 53, 54, 55, 56, 57, 58, 59, 60, 61, 62, 63, 64, 65, 66]. The fact that these microcolumns also have small void times and low backpressures makes them of interest for high‐throughput methods or techniques that require high flow rates or short column residence times [50, 52, 63]. In addition, the ability to often reuse the same binding agent for hundreds of experiments provides µAC with good precision and reproducibility; the same feature, combined with the small amount of binding agent already present, can lead to even less binding agent being needed in µAC per experiment than is required in ACE or related methods [9].

Various supports and column dimensions have been used to create affinity microcolumns and platforms for µAC [9, 60, 62, 67, 68, 69]. Both particulate supports (e.g., silica) and monoliths (i.e., as based on polymethacrylates or silica) have been employed for this purpose. Some of these microcolumns have had lengths of 1–5 cm and an I.D. of 2.1 mm or less (∼35–175 µL total volume) [9]. Affinity discs, with typical lengths of 1–2 mm and an I.D. of 4.6 mm (∼15–35 µL total volume) have also been used [60, 67, 68, 69]. In addition, sandwich columns have been employed that contain affinity layers with thicknesses of 60–250 µm and a I.D. of 4.6 mm (0.2–0.9 µL volume) [62].

In some cases, capillary columns have been used in µAC [9, 47, 49, 53, 54, 55, 56, 57, 58, 62, 63, 69, 70, 71, 72, 73, 74, 75, 76, 77]. This has included open tubular columns with an I.D. of 100 µm and lengths of 30–40 cm (2–4 µL total volumes) [62, 63, 69]. Packed capillaries with an I.D. of 0.5 mm and lengths of 5–15 cm (10–30 µL total volume) have been employed as well in microscale affinity separations [47, 49, 53, 54, 55, 56, 57, 58, 70, 71]. These types of columns are usually utilized in applications that may require the use of low flow rates (i.e., nL/min to µL/min range) or coupling of the affinity support with mass spectrometry [72, 73, 74, 75, 76]. As a result, this type of platform typically requires specialized instrumentation that is compatible with microbore or nanobore LC systems [72, 73, 77].

Microchip and microfluidic systems are another approach for working with microscale affinity separations [52, 78, 79, 80, 81, 82, 83, 84]. This involves the use of binding agents that are coated within microchannels or immobilized onto beads that are then placed within a microchip [78, 79]. Some continuous‐flow microfluidic channels can be categorized under microcolumn formats when they incorporate immobilized affinity ligands on channel walls or packed beads, thus functioning as miniaturized chromatography columns [79]. Examples are platforms such as packed microfluidic channels (e.g., with streptavidin beads and biotin‐labelled ligands) or microcolumns on centrifugal platforms (e.g., with immobilized antibodies against a given target) for on‐chip AC [52, 80, 81]. Such formats have been used for the high‐throughput evaluation of operation conditions in biomolecular separations [83, 84].

## 3 Zonal Analysis in Microscale Affinity Chromatography

One common format in µAC for biointeraction studies is zonal analysis [33, 84, 85]. In this approach, a small band of a target solute or probe compound is applied to a microscale affinity platform that has an immobilized ligand of interest [16, 33]. The application of this band is done under pH, solution, and temperature conditions that promote binding by the target or probe with the immobilized ligand. Any of these conditions can also be varied, including the addition of a competing agent to the mobile phase, to examine the nature of this interaction [33, 85].

In zonal analysis, the retention time (*t*_R_​) of the injected target or probe on an affinity platform containing an immobilized ligand is determined and compared to the void time (*t*_M_). These data are used to obtain the injected solute's retention factor (*k*), as accomplished by using the relationship *k* *=* (*t*_R_ *—t*_M_)*/t*_M_ [33, 85, 86]. If non‐specific binding by the target is also present with the support, the specific retention factor for the target with the binding agent (*k* *′*) can be determined by finding the difference in the retention factors for this target on platforms that are prepared in both the absence versus presence of the immobilized ligand [85, 86].

One way that zonal analysis and retention factor measurements can be used in µAC is to estimate the binding strength of the ligand or the amount of active ligand that is in the affinity platform. For instance, if this experiment is done with a small amount of applied solute and under linear elution conditions, the specific retention factor acquired for an injected target can be related to both the moles of active ligand present (*m*_L_) and the association equilibrium constant for this target and ligand (*K_a_* ​), as demonstrated in Equation (5) [16, 33].

(5) $$k^{\prime }=K_{a\;}\frac{m_{L}}{V_{M}}$$

In this relationship, *V*_M_​ is the void volume of the column. Similar expressions can be written for more complex systems, such as a compound that has a series of independent binding sites on the immobilized ligand [16, 33].

**FIGURE 2:**
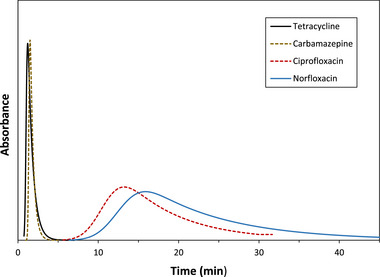
Use of zonal analysis and µAC to examine the binding of several pharmaceutical‐based environmental contaminants to affinity microcolumns containing an entrapped sample of humic acid, a natural form of dissolved organic matter in water. These results were acquired on 10 mm × 2.1 mm I.D. microcolumns at 0.50 mL/min and 25°C in a mobile phase that was pH 7.4, 0.067 M potassium phosphate buffer. These results are from Ref. [90] and adapted with permission from Elsevier.

Recent examples that have used this direct analysis approach in µAC include studies in which microcolumns containing normal or modified human serum albumin (HSA) have been used to examine the binding strength of various antidiabetic drugs with this protein in the presence of modifications such as glycation or advanced glycation end‐products [86, 87, 88, 89]. In addition, microscale affinity columns have been used with entrapped samples of humic acid to study binding by this natural environmental agent with various pharmaceuticals that occur as contaminants in water, as shown in Figure 2 [90, 91]. Measurements of overall affinity by zonal analysis methods have been used with microcolumns containing HSA or alpha_1_‐acid glycoprotein (AGP) to examine the binding and stereoselectivity of these proteins for lofexidine and related substances [92]. Zonal analysis has been employed with injections of *R*‐ and *S*‐propranolol to monitor the activity and stability of microscale affinity columns containing high‐ or low‐density lipoproteins [93, 94, 95]. In addition, microscale affinity columns have been utilized with zonal analysis to see how changes in temperature, ionic strength, and pH affect the interactions of various drugs with serum proteins or humic acid [85, 88, 89, 91, 96].

Zonal analysis with µAC has been frequently used to study competition and displacement of solutes as they take part in drug‐protein binding and other low‐to‐moderate strength processes [33, 85]. In this method, a probe is injected onto an affinity column in a mobile phase that has a known concentration of a target analyte or suspected competing agent [16, 85]. The retention of the probe compound is then measured as various concentrations of the target in the mobile phase are passed through the column. This information is used to determine whether these compounds share the same sites for binding on the ligand or exhibit allosteric effects as they bind to this ligand. This approach can reveal the number and location of interaction sites, when examined with probes for these sites, and allows the strength of binding at specific regions to be measured [33, 85, 87, 88, 89, 97, 98]. For instance, if an injected probe (P) and a target analyte that is present in the mobile phase (A) compete for a single set of sites on an immobilized ligand, Equation (6) will describe the way in which the retention factor for P (*k*_P_) is altered as the molar concentration of A is varied [16, 33, 85].

(6) $$\frac{1}{k_{P}}=\;\;\frac{\left.K_{a,A}[A\right]}{K_{a,P}C_{L}}+\frac{1}{K_{a,P}C_{L}}$$

The terms *K*_a,P_ and *K*_a,A_​ in Equation (6) are the association equilibrium constants of the ligand for the injected probe and target analyte, respectively, and *C*_L_ ​ is the concentration of active ligand in the column. If P and A bind at the same site on the ligand, the slope‐to‐intercept ratio from Equation (6) will provide the value of *K*_a,A_ for A at that site [33, 85].

**FIGURE 3:**
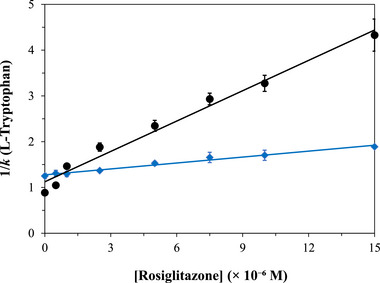
Use of zonal analysis with µAC and Equation (6) to examine the competition of L‐tryptophan (i.e., a probe for binding at Sudlow site II of HSA) with the antidiabetic drug rosiglitazone on affinity microcolumns containing HSA that had been modified with glyoxal (diamonds) or methylglyoxal (circle) to form advanced glycation end‐products. These data were acquired on 10 mm × 2.1 mm I.D. microcolumns at 0.25 mL/min and 37°C in a mobile phase that was pH 7.4, 0.067 M potassium phosphate buffer. The error bars represent ± 1 SD. These results are from Ref. [89] and adapted with permission from Elsevier.

Recent experiments have employed competition‐based studies with zonal analysis and µAC to examine the interactions of cathinones and a variety of antidiabetic drugs with both modified and native forms of HSA (see Figure 3) [87, 88, 89, 96]. Additional applications have included the use of this approach for screening potential arginase inhibitors and anticancer drugs, as well as characterization of the binding by bioactive constituents from traditional medicines to the α_1A_‐adrenoreceptor, voltage dependent anion channel isoform 1 (VDAC‐1), and vascular endothelial growth factor 2 (VEGFR2) [99, 100, 101, 102].

Equation (6) and related equations have been employed to identify the presence of direct competition versus non‐competition or allosteric effects between two solutes and a given ligand [33, 85, 88, 89]. For instance, this evaluation can be done in a quantitative fashion by examining the fit of the retention data to a relationship such as Equation (7) [97].

(7) $$\frac{k_{0}}{k-k_{0}}=\frac{1}{\beta_{I\to A}-1}\;\cdot \;\frac{1}{K_{a,\;\mathrm{IL}}\;\left[I\right]+1}$$

In Equation (7), the retention factors *k*_0_ and *k* are acquired for A in the absence and presence of competing agent I, respectively. Other terms here include *K*_a,IL_, which is the association equilibrium constant for the competing agent at its site of binding on the immobilized ligand, and β_I→A_, which is the coupling constant between the sites at which I and A bind. Equations (6) and (7) and related expressions have been utilized to construct affinity maps describing the multi‐site binding and interactions of antidiabetic drugs with normal and modified HSA using µAC [88, 89].

Another application of zonal analysis with microscale affinity columns has involved studies of the interaction of target analytes with an additional ligand that is present in the mobile phase. For example, a microscale column containing immobilized cellular retinoic acid binding protein (CRABP) was preloaded with retinoic acid, followed by introduction of another potential binding agent, retinoic acid receptor isoform γ (RARγ), that was placed in the mobile phase [103]. A comparison was then made between the elution profile of RARγ on this column and a control column to see if there were interactions between CRABP and RARγ, thus reflecting the transfer of retinoic acid between these two agents [103]. Zonal analysis has been used with mass spectrometry and cyclin G‐associated kinase in packed capillaries to rank the affinities of this agent for drug fragments [104]. The same approach has been applied to examine binding by the chaperone protein HSP90 with various drug fragments [59], for carbohydrates with hen egg‐white lysozyme [105], and for anti‐cancer compounds with VDAC‐1 [99].

The previous applications described for zonal analysis were typically conducted under linear conditions, which occur when the injected solute is present in trace amounts relative to the immobilized target [46, 97, 106, 107, 108]. Although using a large amount of solute makes detection easier, this will quickly lead to non‐linear conditions in which solute retention and peak shape now vary with the solute load [46, 97, 106, 107, 108, 109, 110, 111, 112, 113, 114, 115, 116, 117, 118]. There are several ways of processing data in this situation. The first option is to fit solute peak profiles to an expression such as Equation (8) which may account for these non‐linear effects [109, 110, 114, 115, 116].

(8) $$y=\frac{a_{0}}{a_{3}}\;\left[1-e^{\left(\frac{-a_{3}}{a_{2}}\right)}\right]\;\left[\frac{\left(\sqrt{\frac{a_{1}}{x}}\right)I_{1}\;\left(\frac{2\;\sqrt{a_{1}x}}{a_{2}}\right)\;e^{-x-a_{1}/a_{2}}}{1-T\;\left(\frac{a_{1}}{a_{2}},\frac{x}{a_{2}}\;\right)\;[1-\;e^{-a_{3}/a_{2]}}}\right]$$

The *y* term in this equation is the peak response at reduced retention time *x*; *T* is a switching function; *I*_1_ is a modified Bessel function; and *a*_0_​ through *a*_3_​ are the parameters used to fit Equation (8) to the peak. The best‐fit values for this last set of parameters can be used to determine the association equilibrium constant and apparent dissociation rate constant for the interaction of the solute with the immobilized ligand [46, 114, 115, 116]. This peak‐fitting approach has been used with microscale affinity columns to estimate rate constants for solutes and drugs with endothelin rReceptor A and VDAC‐1, as well as with muscarinic‐3 acetylcholine, cysteinyl‐leukotriene type 1, and α3β2 nicotinic acetylcholine receptors [99, 107, 108, 112, 116]. This method has also been applied to investigate the binding of bronchodilators with β_2_‐adrenoceptor [106, 117, 118].

A second approach that can employ non‐linear conditions with zonal analysis and microscale affinity columns is the injection‐amount dependent method, as represented by Equation (9) [119].

(9) $$\frac{k\;n_{A}}{1+k}=m_{L}-\;\frac{k\;V_{M}}{K_{a}}$$

In this technique, known amounts of the target analyte, as given in this equation by the moles of A (*n*_A_), are introduced into an affinity column with the immobilized ligand. The corresponding retention factor for A (*k*) is measured and then used with *n*_A_ to construct a plot of (*k* *n*_A_)/(1 + *k*) versus (*k* *V*_M_), in which *V*_M_ is again the column void volume. Under ideal conditions, this type of analysis should result in a linear relationship that will provide *K*_a_ for the analyte–ligand interaction and the moles of active ligand binding sites (*m*_L_), as obtained from the slope and intercept [119]. This scheme has been utilized in µAC to determine the binding features of compounds that interact with the calcium‐sensing receptor, to characterize the interaction of rosmarinic acid with cysteinyl leukotriene receptor type 1, and to examine the binding of ATP, NADH, and NADPH to VDAC‐1 [99, 107, 120].

## 4 Frontal Analysis in Microscale Affinity Chromatography

Frontal analysis is another technique that has been used in µAC to provide information on biointeractions [9, 34, 121, 122]. This can be done by applying a continuous solution of the target analyte at a known concentration to the immobilized ligand in an affinity column or platform. As the analyte is applied in this system, it will begin to saturate binding sites on the ligand and excess or unbound analyte will elute from the column [34, 121, 122]. This produces a breakthrough curve, which is usually generated at several applied levels of A. The results are then utilized to obtain information on the strength of binding between A and L, the amount of sites taking part in this binding, and the types of binding that are occurring between A and L [9, 34, 121, 122].

This information can be generated by fitting the frontal analysis data to an appropriate equation and binding isotherm that describes the type of interaction that is being studied [34, 121]. For example, if fast kinetics for association and dissociation are present for a 1:1 binding system, as shown earlier in Equations (1)–(3), the central location of the breakthrough curve will be described by Equation (10) [5, 34, 121, 123].

(10) $$m_{L\mathrm{app}}=\;\frac{m_{\;L}K_{a}\left[A\right]}{\left(1+K_{a}\left[A\right]\right)}$$

In this model, 𝑚_L𝑎𝑝𝑝_ is the moles of target analyte needed to reach the central location of the breakthrough curve at an applied analyte concentration of [A], 𝑚_L_ is the total moles of sites for A on the ligand in the affinity platform, and *K*_a_ is the binding constant for A and L. Related equations can be derived for more complex systems, such as those that involve several groups of binding sites or combinations of different types of interactions [5, 34, 121, 123, 124].

**FIGURE 4:**
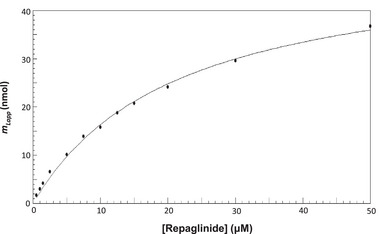
Use of frontal analysis and µAC to examine binding by the antidiabetic drug repaglinide with glycated HSA. These results, as shown with their fit to Equation (10), were obtained using a 1.0 cm × 2.1 mm I.D. microcolumn containing glycated HSA and operated at 0.50 mL/min and 37°C in the presence of pH 7.4, 0.067 M potassium phosphate buffer. These results are from Ref. [87] and adapted with permission from Elsevier.

This general approach has been used with frontal analysis to investigate many types of biointeractions by µAC. For instance, the binding of various antidiabetic drugs with normal or modified HSA has been studied and characterized using this method (see Figure 4) [87, 125]. This form of frontal analysis has further been employed to evaluate binding by 5‐hydroxytryptamine 1A receptor with buspirone, hypidone, and serotonin [126]. In addition, this approach has been utilized with µAC to study the binding and stereoselectivity for *R*‐ and *S*‐propranolol in their interactions with high‐, low‐, and very low‐density lipoproteins [93, 94, 95]. Frontal analysis has further been combined with immunoextraction and microscale affinity columns to screen and measure binding by sulfonylurea drugs with normal HSA and HSA containing advanced glycation end‐products [127].

Another application of frontal analysis with µAC has been in experiments that examine the competition between multiple solutes for a common binding agent [49, 121]. This type of study can be performed by adding the target and a possible competitive agent to the mobile phase, with this mixture then being applied to the ligand in a µAC platform. The variation in the target analyte's breakthrough curve is monitored as the level of the competing agent is altered. If there is direct competition between the target analyte and competing agent, this should lead to a smaller mean breakthrough time for the target as an increase occurs in the competing agent's concentration [9, 48, 49, 121].

This general format for frontal analysis and µAC has often been used with mass spectrometry. This combined method is referred to as frontal affinity chromatography‐mass spectrometry, or FAC‐MS [54, 56, 57, 58, 75, 128, 129, 130, 131, 132, 133, 134]. As an example, FAC‐MS has been utilized to study the interaction of solutes with ATP during their binding with the enzyme protein kinase C [130], as well as to examine the binding of dihydrofolate reductase to possible inhibitors of this enzyme [57]. In addition, this method has been employed to evaluate the catalytic activity of *N*‐acetylglucosaminyltransferase V, along with the binding of inhibitors to dihydrofolate reductase [57, 58]. This approach has also been used in examining carbohydrate–lectin interactions, such as the binding of sialyl‐lacto‐*N*‐tetrose with a lectin from *Polyporus squamosus* [134].

Another form of frontal analysis in µAC is when stepwise application is used to pass various solutions of the target analyte through an affinity platform [135, 136, 137]. This is done to reduce the time involved when using washing steps between separate analyte solutions, as is usually done in traditional studies based on frontal analysis [138, 139]. This modified format produces a ladder‐shaped series of frontal analysis profiles that are used to determine the binding constant for the analyte with the ligand in the column [135, 136, 138]. Stepwise frontal analysis has been utilized to examine drug–protein interactions, such as the binding of immobilized HSA with digitoxin or warfarin and the binding of AGP with tamsulosin or verapamil [135, 136].

## 5 Kinetic Methods in Microscale Affinity Chromatography

µAC has been used in many formats to conduct kinetic studies of biointeractions. One group of such tools include the plate height method and the peak profiling approach, which are both based on measurements of band‐broadening [46, 140, 141]. The plate height method examines the various contributions to band‐broadening for a target solute on an affinity column. The goal of this process is to obtain the plate height term *H_s_*, which represents stationary phase mass transfer. This term can be related to the target's dissociation rate constant (*k*_d_) with the immobilized ligand through Equation (11) [46, 141, 142, 143].

(11) $$H_{s}=\frac{2uk}{k_{d}{\left(1+k\right)}^{2}}\;$$

In this equation, u is the mobile phase's linear velocity, and *k* is the retention factor of the target analyte in the affinity platform. A plot of $H_{s}$ versus $uk/{\left(1+k\right)}^{2}$ is then made, which provides $k_{d}$ from the slope [66, 143, 144].

The plate height method was originally used in HPAC with short, but more traditional‐sized HPLC columns to examine the chiral separation and binding of D‐ or L‐tryptophan and *R‐* or *S*‐warfarin by HSA, in addition to the binding of sugars with the lectin concanavalin A [66, 140, 143]. This technique has also been used to examine how variations in pH, solvent polarity, temperature, and ionic strength affect solute interactions with HSA columns [66]. This technique was later applied in µAC with microscale and monolithic affinity columns to study the binding strength and kinetics of compounds such as carbamazepine, L‐tryptophan, and *R*‐warfarin with HSA [38, 145]. This method has been shown to be well‐suited for measuring *k*_d_ values in the range of ∼10^−2^ to 10^−1^ s^−1^ and in work with interactions that have a moderate‐to‐weak binding strength (i.e., $K_{a}\;$≤ 10^6^ M^−1^) [38, 46, 66, 143, 145, 146].

**FIGURE 5:**
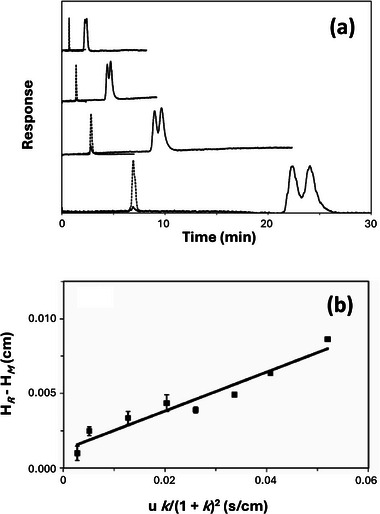
Use of µAC and peak profiling to examine the kinetics for interactions by the protein HSA with two chiral forms of the drug metabolite 5‐(3‐hydroxyphenyl)‐5‐phenylhydantoin (or *m*‐HPPH, for which the parent drug is phenytoin). The chromatograms in (a) were obtained for injections of *m*‐HPPH (solid lines) or sodium nitrate (dashed lines, a non‐retained solute) made at flow rates (bottom‐to‐top) of 0.25, 0.50, 1.00, and 2.00 mL/min and at pH 7.4 and 37°C on a 10 cm × 4.6 mm I.D. microcolumn containing HSA. The data in (b) are for the second eluting enantiomer of *m*‐HPPH, as plotted according to a modified form of Equation (11). These results are from Ref. [154] and adapted with permission from Elsevier.

A related kinetic method is peak profiling [35, 147, 148, 149, 150, 151, 152, 153, 154]. This approach requires the use of measurements at fewer flow rates than the plate height method and allows for a more direct determination of $k_{d}$ [35, 148]. This technique involves determining the retention times and total plate heights of the target analyte under linear elution conditions on both a control column and an affinity column containing the desired ligand. The difference in these plate height values is then utilized to provide $H_{s}$ and $k_{d}$ (see Figure 5) [35, 147, 149, 154].

The peak profiling method was originally used with standard‐sized LC columns to study the self‐association of bovine neurophysin II and its interaction with neuropeptides such as Arg^8^‐vasopressin [46, 141, 146]. It was later used with HPAC to examine the dissociation kinetics of HSA with *R‐* or *S*‐warfarin and D‐ or L‐tryptophan [66, 109, 144, 148]. More recently, peak profiling has been employed with µAC to examine such systems as HSA in its binding with imipramine, carbamazepine, and L‐tryptophan and the binding of AGP with drugs such as chlorpromazine and verapamil. Other examples have been the utilization of this approach to study the binding of β‐cyclodextrin with acetaminophen and sertraline and to examine the interactions of β_2_‐adrenoceptor with salbutamol and ephedrine hydrochloride [46, 117, 151, 152, 153]. This technique has also enabled simultaneous analysis of the dissociation kinetics for chiral metabolites of phenytoin with HSA, as shown in Figure 5 [154]. The approach has further been used to simultaneously determine $k_{d}$ for drugs like acetaminophen, trimethoprim, and *S*‐flurbiprofen with β‐cyclodextrin [152, 153].

**FIGURE 6:**
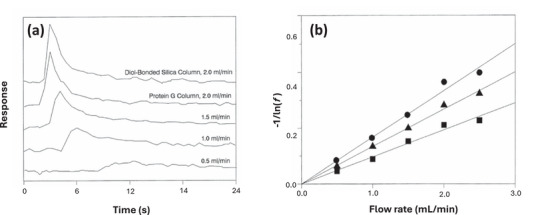
Use of µAC with the split‐peak method to examine the rate of association by immunoglobulin‐binding proteins (protein A and protein G) with injected samples of immunoglobulin G (IgG). The results in (a) compare the non‐retained peaks seen for rabbit IgG that was injected at several flow rates on microcolumns that contained either a control support (diol‐bonded silica) or a support with immobilized protein G. The graphs in (b) are plots that were made according to Equation (12) for the measured free fractions of IgG on columns containing protein G (top), protein G + protein A (middle), or protein A (bottom). These results were acquired on 6.35 mm × 2.1 mm I.D. microcolumns. These figures are from Ref. [156] and adapted with permission from Elsevier.

The split‐peak method is another approach that relies on µAC for examining the rates of biomolecular interactions [9, 41, 155]. This technique is based on the “split‐peak effect,” a phenomenon in which a small amount of an applied target analyte can pass through a column without being retained by an immobilized ligand, even if the amount of this target is well below the column's binding capacity. This effect, as illustrated in Figure 6 [156], is enhanced as either the flow rate is increased or the column size is decreased, thereby lowering the target's time of residence in the column. For example, the extent of this effect when the amount of target is small compared to the amount of ligand is described by Equation (12),

(12) $$-\frac{1}{\ln\left(f\right)}=F\left(\frac{1}{k_{m}V_{e}}+\frac{1}{k_{a}m_{L}}\right)$$

in which f is the free (or non‐retained) fraction of the target analyte and $k_{a}$ is the association rate constant for this target with the immobilized ligand [43, 46, 142]. Other terms in this equation are the flow rate (*F*), the column's excluded volume ($V_{e}$), the target's mass transfer rate constant as it moves into the pores or stagnant mobile phase region of the support ($k_{m}$), and the total moles of immobilized ligand that are present ($m_{L}$). According to Equation (12), if −1/ln(f) is plotted against *F* under linear elution conditions, the line that is obtained should have a slope that is related to both the rate of stagnant mobile phase mass transfer, $1/(k_{m}V_{e})$, and the rate of analyte adsorption to the stationary phase, $1/(k_{a}m_{L})$. This slope further simplifies to the second of these terms if the binding of analyte to the ligand is the rate‐limiting step in the retention process, making it then possible to estimate $k_{a}$ [9, 41, 43, 46]. Related expressions have been derived and reported for use under non‐linear conditions for systems in which target adsorption is the slow step in retention [155, 156, 157, 158, 159].

The split‐peak method was originally used to study the adsorption kinetics of rabbit IgG on microscale columns containing protein A [43]. This technique was also utilized to help design µAC protein A columns for the measurement of IgG in serum [32]. The same method was later extended to investigate the binding rates of IgG with protein G and mixed protein A/G supports (see Figure 6) [43, 156]. In other reports, this technique has been applied under non‐linear elution conditions to obtain association rate constants in various systems, including the binding of HSA and 2,4‐dichlorophenoxyacetic acid (2,4‐D) with antibodies against these targets, as well as the binding of L‐thyroxine with aptamers developed for this hormone [46, 159, 160, 161]. The *k*_a_ values that have been measured with this approach have spanned from $10^{4}$ and $10^{6}\;M^{-1}\;s^{-1}$ [43, 141]. This method has been found to be best suited for processes with slow dissociation and strong binding, which aids in the production and observation of the split peak effect [43, 156, 159, 160, 161].

**FIGURE 7:**
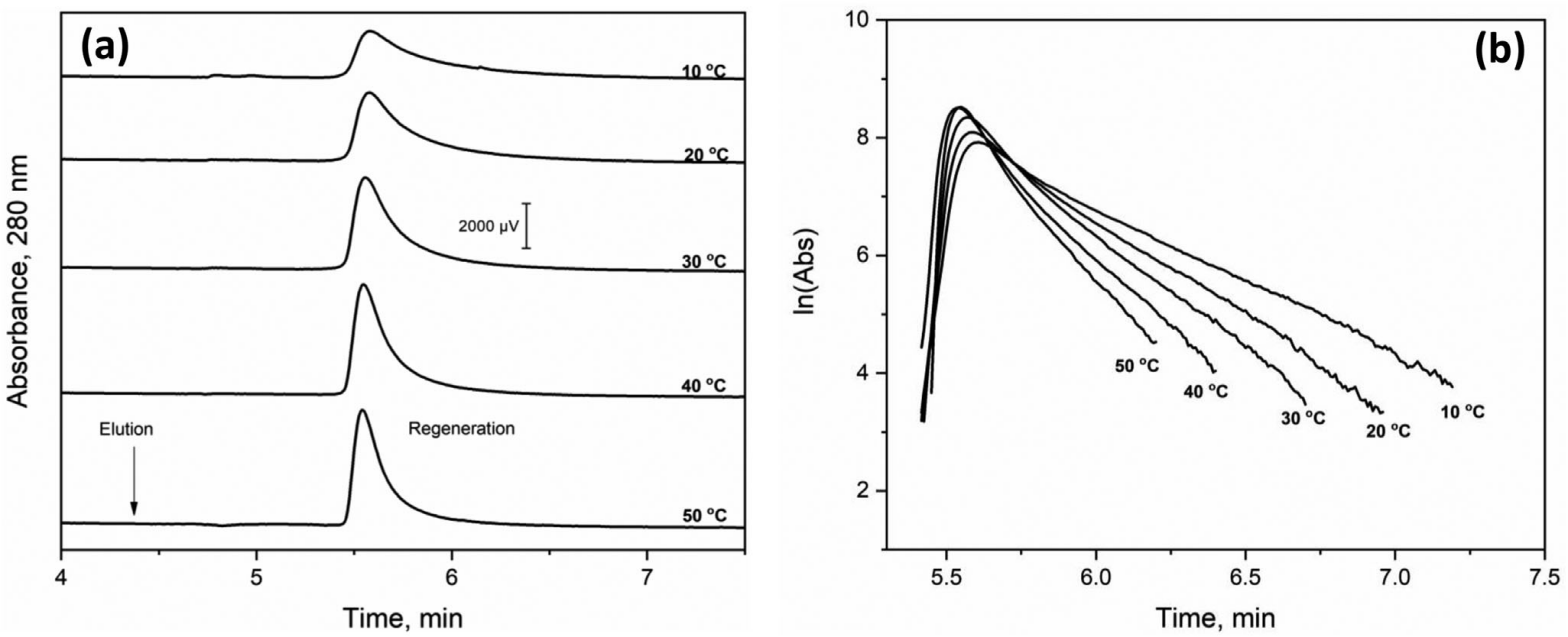
Utilization of peak decay analysis and µAC to study the elution of AGP from a 50 mm × 2.1 mm I.D. microcolumn containing *Aleuria aurantia* lectin, as examined at various temperatures. The plots in (a) show the chromatograms for the elution of AGP and (b) gives the natural logarithms of these elution profiles. These results were obtained at 0.75 mL/min using an application buffer that consisted of pH 7.4, 0.010 M Tris‐HCl buffer plus 0.15 M NaCl and an elution buffer with the same composition but also containing 0.0020 M L‐fucose as a competing agent to promote the elution of AGP. These figures are from Ref. [163] and used with permission from Elsevier.

The peak decay method is an approach in µAC for examining the dissociation rates of biointeractions [9, 41, 141, 162]. The target analyte is applied in this method to a microscale affinity platform containing the ligand of interest. Conditions are then created to promote release of the bound analyte while also minimizing its rebinding to the ligand. One way this can be accomplished is by applying a competing agent that will also bind to the ligand, thereby preventing any dissociated analyte from rebinding. An alternative approach is to inject a large amount of analyte that saturates the ligand and minimizes the chance for the dissociated target to undergo rebinding. An elevated flow rate and small column size are employed in both schemes to further avoid rebinding by the target [41]. As shown in Figure 7, these conditions result in a decay curve for the target as it is released from a µAC column, which can then be utilized to obtain *k*_d_ for the analyte and ligand [41, 46, 142, 143].

The peak decay method was initially used in HPAC to determine the dissociation rates of 4‐methylumbelliferyl α‐D‐mannopyranoside from concanavalin A, as accomplished by using a competing sugar (i.e., 4‐methylumbelliferyl α‐D‐galactopyranoside) to prevent rebinding of the target [46, 162]. It has also been used recently in µAC to study dissociation of the glycoprotein AGP from affinity microcolumns containing *Aleuria aurantia* lectin and in the presence of L‐fucose as a competing agent (see Figure 7) [163]. This method has been further employed for a wide array of other systems, including the determination of *k*_d_ values for drugs such as diazepam, imipramine, acetohexamide, tolbutamide, amitriptyline, quinidine, verapamil, nortriptyline, lidocaine, and racemic warfarin with the serum proteins AGP and HSA [164, 165, 166]. This method has also been used to evaluate the release of L‐thyroxine from antibodies or aptamers against this target, and the dissociation kinetics of IgG from protein G or 2,4‐D from antibodies against 2,4‐D [157, 160, 161]. This method has been reported to be well‐suited for examining processes with moderate or weak binding and with *k*_d_ values spanning from $10^{-2}$ to $10^{1}\;s^{-1}$ [46, 141, 160, 165, 166]. It has also been shown to be valuable in optimizing elution protocols in HPAC and µAC for systems with strong binding, such as those involving antibodies or aptamers [157, 161].

## 6 Ultrafast Affinity Extraction and Microscale Affinity Systems

**FIGURE 8:**
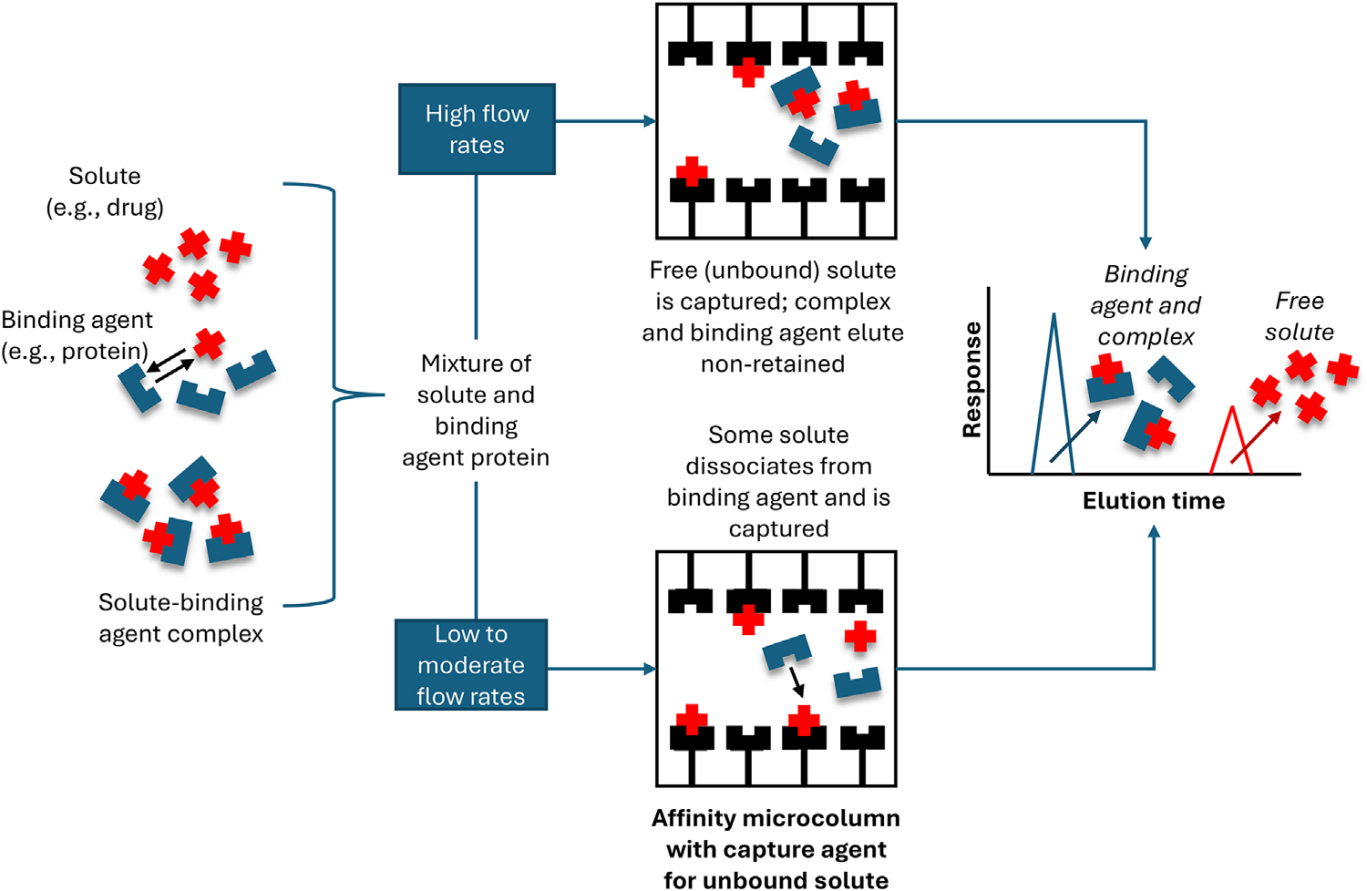
General scheme for ultrafast affinity extraction (UAE) to either measure binding constants at high flow rates or both binding constants and dissociation rate constants at low‐to‐moderate flow rates on a µAC platform.

Ultrafast affinity extraction (UAE) is an additional way in which µAC can be used to examine biomolecular interactions [50, 51, 62, 63, 167, 168, 169]. In this method, a target analyte or solute is injected, either individually or in combination with a binding agent in the sample, onto a microscale affinity platform that contains a secondary ligand to capture the target analyte in its free or unbound form (see Figure 8) [168, 169]. Selection of this secondary ligand will depend on the intended application and may range from highly specific agents such as antibodies to more general binding partners (e.g. HSA or AGP) [1, 50, 62, 64, 169, 170]. The column size and flow rate used for this process are selected to typically create residence times in the column for the binding agent and non‐retained sample components that are in the millisecond‐to‐second scale. These conditions make it possible to control and/or minimize the extent of dissociation of the target analyte from the binding agent in the sample as this mixture travels through the µAC platform [50, 52, 64, 167, 171]. This, in turn, allows the captured target to be used in estimating the unbound fraction of this compound in the original sample [50, 51, 62, 63, 167, 168, 169, 170].

The measured free fraction that is acquired by UAE can be used to estimate both the binding constant and dissociation rate of the target analyte from the soluble binding agent [50, 51, 62, 63, 167, 168, 169, 170]. For example, the free fraction of the target analyte that is measured at a short column residence time *t* (*F_t_*), such as obtained when using a high flow rate, should approach the true free fraction (*F*_o_) for the same target at equilibrium and in its mixture with the soluble binding agent. The value of *F*_o_ can then be used to obtain the association equilibrium constant (*K*_a_) for the target with the same binding agent by using a relationship like the one in Equation (13) for a system with 1:1 binding for A with L [50, 52, 167, 168, 172, 173, 174, 175, 176].

(13) $$K_{a}=\frac{1-F_{o}}{F_{0}\left(\left(C_{L}\right)-\left(C_{A}\right)+\left(C_{A}\right)F_{o}\right)}\;$$

In Equation (13), C_A_ is the total concentration of analyte in the sample, and C_L_ is the total concentration of the soluble binding agent. If *F_t_* is instead measured at several flow rates (i.e., spanning low‐to‐moderate values) and over various column residence times, Equation (14) can be used to plot these data to find both the value of *F*_o_ and the dissociation rate constant (*k*_d_).

(14) $$\ln\;\frac{1}{\left(1-F_{t}\right)}=k_{d}\;t-\ln\left(1-F_{o}\right)$$

One way this can be done is by making a plot of ln[1/(1 − *F_t_*)] versus *t* and using the best‐fit intercept and slope from the linear response of this graph to estimate *k*_d_ and 𝐹_o_ [168].

UAE has been used in µAC with both single‐ and multi‐column systems to investigate biointeractions and measure the free fractions for drugs, hormones, and other solutes in the presence of various binding agents. For example, this method has been used to examine the binding of warfarin with normal or modified forms of HSA [52, 175, 177]. This approach has further been used to evaluate binding by HSA, equine serum albumin, and sex hormone binding globulin with testosterone, to examine the interactions of various sulfonylureas with normal or modified HSA, and to study binding by numerous drugs with AGP [50, 51, 63, 64, 167, 168, 170, 171, 172, 173, 174, 175, 176, 178].

Some advantages of UAE include its need for only small sample volumes, its fast analysis times, and its ability to examine binding without the need for immobilization of either the target analyte or binding agent of interest [50, 51, 167, 168, 171, 172, 173, 176]. This approach has been used with various detection methods, including label‐free schemes based on direct absorbance or fluorescence measurements of the eluting target. UAE has also been combined with chromatographic immunoassays and with the use of chemiluminescence, fluorescence, and near‐infrared fluorescence for the measurement of target analytes that occur at trace levels in their free fractions [50, 51, 168, 171, 173, 176].

## 7 Concluding Remarks

This review has presented an overview of how µAC can be employed as a tool to examine the strength and rates of biointeractions. Approaches that were discussed for the measurement of binding strength or to examine competition of target analytes for the same binding ligand included a variety of methods based on zonal or frontal analysis. Zonal analysis is often used for binding studies in situations where only a small amount of a target analyte is available or multiple retained components are present in a sample [1, 2]. This method is also useful if the goal is to screen the overall binding of targets to an immobilized binding agent; to see how this binding varies with temperature or solution conditions; or to use probes to carry out site‐specific binding and competition studies with the target [1, 2, 3, 33, 85]. Frontal analysis is often employed when the amount of target is less of concern and more detailed information is needed on the general types and amounts of binding regions that may be present for a target analyte with the affinity ligand [1, 2, 34, 122]. In addition, frontal analysis tends to be more commonly used than zonal analysis with mass spectrometry to screen the binding of various targets with an immobilized agent [2, 54, 56, 57, 58, 75, 128, 129, 130, 131].

It was also shown how kinetic studies could be conducted with techniques such as band‐broadening measurements, the split‐peak method, and the peak decay method. Some of these methods (e.g., band‐broadening techniques) can be used under the same conditions as employed with zonal or frontal analysis to examine the binding of a target analyte with an immobilized agent [1, 2, 46] and are typically used for systems with relatively fast kinetics and moderate‐to‐strong binding [45, 46, 141, 142]. Other approaches, such as the split‐peak or peak decay analysis methods, require more specialized conditions (e.g., short column residence times) to examine the association and dissociation constants of targets with their binding agents [141, 142, 155, 162]. The choice of these methods will depend on the general range of rate constants and binding affinities that may be present in the system to be studied [141, 142, 162].

The use of immobilized ligands as secondary capture agents to examine a biointeraction, as employed in UAE, was described as well [141, 142]. This method can be used in situations where it is desirable to examine the interactions between a target analyte and its binding agent in a short time frame and directly in solution [2, 141, 142, 167, 168]. The conditions used in this method can be varied to allow both binding constants and dissociation rate constants to be obtained [141, 142]. However, UAE requires use of a µAC column with a suitable capture agent for one of the interacting components and which can be used to extract this component on a short time scale, typically in the millisecond‐to‐second range [50, 52, 64, 167, 168].

The information and examples of applications that were provided in this review should allow for the future extension of these methods to other biointeraction systems. This should promote the further development and use of these techniques and µAC for the analysis of biochemical and chemical interactions. It is expected, in turn, that µAC will then see even greater use for this type of application in fields such as biochemical, clinical, and environmental research.

## Author Contributions

**David S. Hage**: conceptualization, funding acquisition, formal analysis, visualization, supervision, project administration, writing – review and editing. **Nigar Sultana Pinky**: investigation, formal analysis, writing – original draft. **B. K. Sajeeb**: investigation, formal analysis, writing – original draft. **Md. Masudur Rahman**: investigation, formal analysis, writing – original draft. **Harshana Olupathage**: investigation, formal analysis, writing – original draft. **Samiul Alim**: investigation, formal analysis, writing – original draft. **Isaac Kyei**: investigation, formal analysis, writing – original draft. **Zoe Zingler**: investigation, formal analysis, writing – original draft. **Sanduni Heenkenda**: investigation, formal analysis, writing – original draft.

## Conflicts of Interest

The authors declare no conflicts of interest.

## Data Availability

Any new data presented in this review is available upon request to the authors.
